# Autophagy and chemotherapy resistance: a promising therapeutic target for cancer treatment

**DOI:** 10.1038/cddis.2013.350

**Published:** 2013-10-10

**Authors:** X Sui, R Chen, Z Wang, Z Huang, N Kong, M Zhang, W Han, F Lou, J Yang, Q Zhang, X Wang, C He, H Pan

**Affiliations:** 1 Department of Medical Oncology, Sir Run Run Shaw Hospital, Zhejiang University, Hangzhou, China; 2 Department of Colorectal Surgery, Sir Run Run Shaw Hospital, Zhejiang University, Hangzhou, China; 3 Department of Gastrointestinal Surgery, Zhejiang Provincial People's Hospital, Hangzhou, China; 4 Biomedical Research Center and Key Laboratory of Biotherapy of Zhejiang Province, Hangzhou, China

**Keywords:** autophagy, chemotherapy resistance, cancer, therapy

## Abstract

Induction of cell death and inhibition of cell survival are the main principles of cancer therapy. Resistance to chemotherapeutic agents is a major problem in oncology, which limits the effectiveness of anticancer drugs. A variety of factors contribute to drug resistance, including host factors, specific genetic or epigenetic alterations in the cancer cells and so on. Although various mechanisms by which cancer cells become resistant to anticancer drugs in the microenvironment have been well elucidated, how to circumvent this resistance to improve anticancer efficacy remains to be defined. Autophagy, an important homeostatic cellular recycling mechanism, is now emerging as a crucial player in response to metabolic and therapeutic stresses, which attempts to maintain/restore metabolic homeostasis through the catabolic lysis of excessive or unnecessary proteins and injured or aged organelles. Recently, several studies have shown that autophagy constitutes a potential target for cancer therapy and the induction of autophagy in response to therapeutics can be viewed as having a prodeath or a prosurvival role, which contributes to the anticancer efficacy of these drugs as well as drug resistance. Thus, understanding the novel function of autophagy may allow us to develop a promising therapeutic strategy to enhance the effects of chemotherapy and improve clinical outcomes in the treatment of cancer patients.

## Facts

- The induction of autophagy in response to metabolic and therapeutic stresses can have a prodeath or a prosurvival role, which contributes to the anticancer efficacy of these drugs as well as drug resistance.
- Anticancer drugs induce different effects of autophagy on cell survival in different cancer types.
- Autophagy as a prosurvival and resistance mechanism against chemotherapy treatment.
- Autophagy-mediated cell death mechanism contributes to efficacy of anticancer drugs.
- Targeting autophagy will hopefully provide a promising therapeutic strategy to circumvent resistance and enhance the effects of anticancer therapies for cancer patients.

## Open Questions

- Whether we should try to enhance or inhibit autophagy in cancer treatment?
- Chloroquine and its derivative: just act as autophagy inhibitors?
- Autophagy is shown to precede apoptosis or act in parallel with this cellular process in addition to be an alternative mechanism to cell death when apoptosis is inhibited. Therefore, the autophagy induction may exert other possibilities, which should be considered in the design of new treatments for the malignancies.

**Figure 1:**
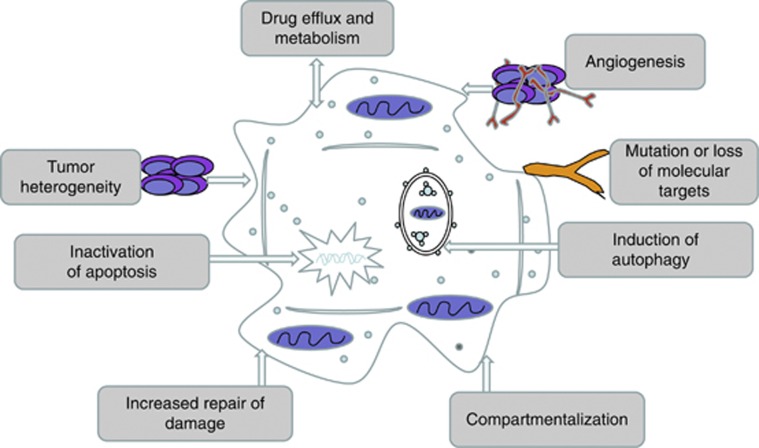
A summary of the approaches by which cancer cells become resistant to chemotherapy and various kinds of genotoxic or metabolic stresses

Resistance to anticancer drugs is a common clinical issue in the treatment of patients with cancer. Drug resistance, intrinsic or acquired, can be attributed to a wide variety of mechanisms including tumor cell heterogeneity, drug efflux and metabolism and tumor microenvironment stress-induced genetic or epigenetic alterations as a cellular response to drug exposure (Figure 1).^1, 2^ Among these mechanisms, the response or adaptation of cancer cell itself to anticancer drug-induced tumor microenvironment stresses is a vital cause for chemotherapy resistance.

Autophagy is an evolutionarily conserved catabolic process in which portions of cytosol and organelles are sequestered into a double-membrane vesicle and delivered to the lysosome for bulk degradation.^3, 4, 5, 6^ In this review, the term ‘autophagy' refers to macroautophagy. The role of autophagy in regulating cancer cell death or survival remains controversial. Current evidence supports the idea that constitutive autophagy can act as a cellular housekeeper to eliminate damaged organelles and recycle macromolecules, thus protecting against cancer, particularly during malignant transformation and carcinogenesis. In established tumors, autophagy can function as a prosurvival pathway in response to metabolic stresses such as nutrient deprivation, hypoxia, absence of growth factors and the presence of chemotherapy or some targeted therapies that might mediate resistance to anticancer therapies.^7, 8, 9^ In this review we will summarize the possible role of autophagy as a novel target for anticancer therapies and discuss the attractive prospect of manipulating this control as a revolutionary strategy for cancer therapy.

## The Regulation of Autophagy in Cancer During Response to Multiple Stresses

Autophagy is essential for not only cell survival but also organism survival in response to microenvironmental stresses. When cancer cells are subjected to stressful conditions, autophagy is rapidly upregulated to maintain metabolic homeostasis and ensure that cell growth is appropriate to its changing environmental conditions through reduced growth and increased catabolic lysis of excessive or unnecessary proteins and organelles. However, persistent or excessive autophagy is also shown to promote cell death following treatment with specific chemotherapeutic agents, either by enhancing the induction of apoptosis or mediating ‘autophagic cell death'.

**Figure 2:**
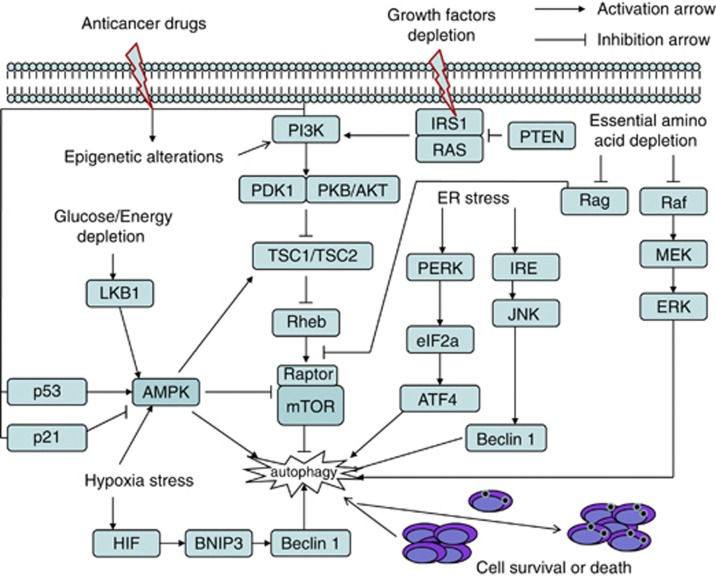
Interrelations between autophagy-related signaling and cell growth control in response to stress. Autophagy can be activated in response to multiple stresses during cancer progression, including nutrient deprivation, endoplasmic reticulum stress, hypoxia, glucose/energy depletion, chemotherapy and other diverse stresses. The AMPK/mTOR pathway functions as a central conduit for autophagic signaling pathways to promote cell survival or death

Although the molecular mechanisms whereby autophagy mediates its effects on both normal and cancer cells are far from complete, various signaling pathways have been implicated in the upregulation or downregulation of autophagy.^10, 11^ The phosphatidylinositol 3-kinase/mammalian target of rapamycin (PI3K/mTOR) and AMP-activated protein kinase (AMPK) signaling pathways have emerged as the central conduit in the regulation of autophagy (Figure 2). mTOR can be activated by growth factors signal through the class I PI3K/Akt pathway, and inhibited by AMPK and p53.^12, 13^ Once activated, mTOR exerts a negative effect on autophagy by phosphorylating a complex of autophagy proteins (ULK1/2), which inhibits the downstream autophagy cascade.^14, 15^ In contrast, AMPK can suppress mTORC1 signaling to stimulate autophagy through TSC1/2 phosphorylation.^16, 17^ Several of the known tumor-suppressor genes (*p53*, *PTEN*, *TSC1/TSC2*) and tumor-associated genes (*p21*, *AKT*) also respectively stimulate or inhibit autophagy.^10, 15^

Autophagy is also induced by a variety of metabolic stresses such as endoplasmic reticulum (ER) stress, hypoxia, oxidative stress, expression of aggregate-prone proteins, glucose deprivation and so on.^18^ ER stress stimulates autophagy through the PERK/eukaryotic initiation factor 2*α* (eIF2*α*) and IRE1/JNK1 pathways. PERK/eIF2*α* phosphorylation has been shown to be essential for the transcription of key autophagy-associated genes during ER stress and may mediate the polyglutamine-induced LC3 conversion.^19^ The activation of IRE1/JNK promotes phosphorylation of Bcl-2 and p53, resulting in interfering with Bcl-2 binding to Beclin 1 and autophagic cell death in cancer cells.^20^ Depletion of nutrients or energy induces autophagy by activating the AMPK pathway or promoting upregulate transcription of certain autophagy genes.^16, 17^ The MEK/ERK signaling activation and Rag inactivation contribute to amino acid depletion-induced autophagy.^7, 21^ Many anticancer drugs including novel targeted therapies stimulate autophagy by inhibiting the PI3K/Akt/mTOR axis or altering genetic/epigenetic phenotype of cancer cells, which provides a survival advantage for struggling tumor cells.^22, 23, 24^ The histone deacetylase (HDAC) inhibitors are recently involved in the control of DNA damage response (DDR) and autophagy. SD118-xanthocillin X (1), a novel marine agent extracted from Penicillium commune, induces autophagy through the inhibition of the MEK/ERK pathway.^25^ Overall, autophagy is a cell biological process that involves diverse signals that have overlapping functions in autophagy and the control of other cellular stress responses.

## Autophagy in Response to Chemotherapy

**Figure 3:**
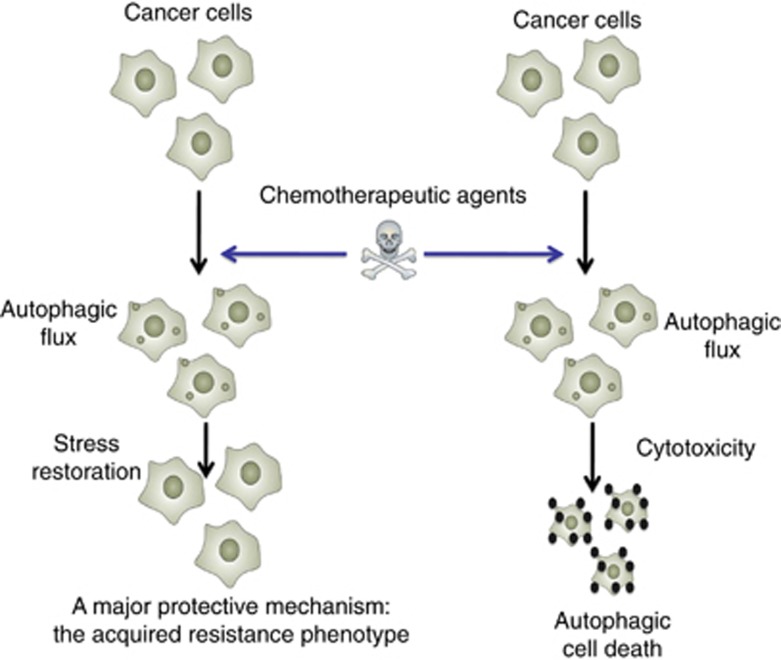
Dual role of autophagy for therapeutic purposes in cancer. On one hand, autophagy is activated as a protective mechanism to mediate the acquired resistance phenotype of some cancer cells during chemotherapy. On the other hand, autophagy may also function as a death executioner to induce autophagic cell death, a form of physiological cell death that is contradictory to apoptosis

Similar to its potential to either induce cell death or promote cell survival, a growing body of evidence implicates a paradoxical role of autophagy following anticancer treatments, with response increasing or diminishing their anticancer activity. On the one hand, autophagy is activated as a protective mechanism to mediate the acquired resistance phenotype of some cancer cells during chemotherapy. Thus, the inhibition of autophagy can re-sensitize previously resistant cancer cells and augment cytotoxicity of chemotherapeutic agents. On the other hand, autophagy may also play as a death executioner to induce autophagic cell death, a form of physiological cell death which is contradictory to type I programmed cell death (apoptosis) (Figure 3). Based on current genetic and pharmacological studies, it appears that anticancer drugs induce different effects of autophagy on cell survival in different cancer types (Table 1). Here we delineate the possible role of autophagy as a novel target for anticancer therapy.

## Autophagy as a Prosurvival and Resistance Mechanism Against Chemotherapy Treatment

Recent studies have demonstrated that tumor resistance to anticancer therapies including radiation therapy, chemotherapy and targeted therapies can be enhanced through upregulation of autophagy in different tumor cell lines.^26, 27^ Moreover, increasing evidence suggests that autophagy inhibition augments cytotoxicity in combination with several anticancer drugs in preclinical models.^28, 29, 30^ Several pharmacological compounds and strategies have been reported to inhibit autophagy *in vitro* and *in vivo*.

## Antimalarial Drugs

The only autophagy inhibitors whose effectiveness *in vivo* and safety in clinical trials have been approved by the FDA are chloroquine (CQ) and its derivative hydroxychloroquine (HCQ) that suppress autophagy by blocking autophagosome fusion and degradation.^31, 32^ Both CQ and HCQ have been investigated in preclinical studies or clinical trials. In comparison with CQ, HCQ can be safely dose escalated in cancer patients.^33^ Currently, more than 30 phase I/II cancer clinical trials (http://clinicaltrials.gov/) involving CQ or HCQ are open around the world and many of them have evidence of preliminary antitumor activity (Table 2).

### Breast cancer

The role of autophagy in breast cancer is an area of active investigation. There is evidence to suggest that epirubicin (EPI) may induce autophagy in human breast cancer MCF-7 cells, resulting in protecting MCF-7 cells from EPI-induced apoptosis.^34^ Autophagy is also regarded as a key mechanism of antiestrogen resistance, and blocking autophagosome can significantly reduce the emergence of antiestrogen-resistant breast cancer cells.^35^ A phase II clinical trial (NCT01292408) is investigating the effects of autophagy inhibition via HCQ on breast cancer patients. The current reports indicate that CQ or HCQ is often used in combination with chemotherapeutic drugs to enhance the efficacy of tumor cell killing; however, its sensitizing effects can also occur independently of autophagy inhibition, which should be considered in the ongoing clinical trials where CQ or HCQ are used in the treatment of breast cancer.^36^

### Colorectal cancer

So far, 5-fluorouracil (5-FU), together with other drugs such as oxaliplatin, remains a widely used chemotherapeutic drug in the treatment of a variety of colorectal carcinomas. Previous researches have demonstrated that inhibition of autophagy augments anticancer effects of chemotherapy or some targeted therapies in colorectal cancer.^37, 38^ Recently, it has been shown that mitogen-activated protein kinase 14 (MAPK14)/p38*α* is involved in resistance of colon cancer cells to 5-FU and irinotecan, which triggers survival-promoting autophagy to protect tumor cells against the cytotoxic effects of these drugs.^39, 40^ Furthermore, autophagy inhibitor CQ significantly enhances the 5-FU-induced inhibition of tumor growth both *in vitro* and *in vivo*.^41, 42^ In addition, the combination of FOLFOX/bevacizumab with HCQ is currently being investigated.

### Esophageal cancer

The role of autophagy in response to chemotherapy and radiotherapy has been investigated in human esophageal squamous carcinoma cells. Autophagy might play a role as a self-protective mechanism in chemotherapeutic drug-treated esophageal cancer cells, and its inhibition has the potential to improve the efficacy of chemotherapeutic agents such as cisplatin and 5-FU.^43, 44, 45^ However, the effect of adding HCQ to conventional therapy for esophageal cancer patients remains unclear.

### Glioblastoma

Malignant cell clones resistant to chemotherapy is a key reason for treatment failure in patients with (GBM). When treated with bevacizumab alone, human GBM xenografts show increased autophagic flux and hypoxia-associated growth, which indicates that hypoxia-mediated autophagy promotes tumor cell survival and resistance to antiangiogenic therapy. However, in treatment by combining with the autophagy inhibitor CQ, tumor growth is disrupted, which elucidates a novel mechanism of overcoming resistance to antiangiogenic therapy for GMB.^46^ A randomized, double-blind, placebo-controlled trial examines the effect of adding CQ to conventional therapy for GBM. As a result, a median overall survival is 24 months for CQ-treated patients and 11 months for placebo-treated patients.^30^ Although it is not statistically different because of the small sample size, the rate of death of patients receiving CQ is prominently lower than controls. Another phase I/II trial concerning dose-limiting toxicities of HCQ with temozolomide (TMZ) and radiation for GBM patients was conducted.^47^ The information about the antitumor activity of this combination is underway.

### Hepatocellular carcinoma

The combination of autophagy inhibitor and chemotherapy or molecular-targeted therapies has been regarded as a promising therapeutic strategy in the treatment of hepatocellular carcinoma (HCC). Autophagy is functionally activated in HCC cell lines after oxaliplatin treatment, and suppression of autophagy enhances oxaliplatin-induced cell death.^48^ Autophagy also contributes to HCC cell survival, and the combined treatment of an autophagy inhibitor and bevacizumab markedly inhibits the growth of HCC.^49^ Moreover, the combination of sorafenib with CQ can generate more ER stress-induced cell death in HCC both *in vivo* and *in vitro*.^50^ Therefore, autophagy inhibition may be a promising therapeutic strategy to enhance the effects of chemotherapy and improve clinical outcomes in the treatment of HCC.

### Leukemia and MCL

Recently, it is reported that high-mobility group box 1 (HMGB1), a damage-associated molecular pattern (DAMP) molecule, contributes to chemotherapy resistance though the upregulation of autophagy in leukemia.^51, 52^ Autophagy inhibitor HCQ is also shown to decrease cell viability of B-chronic lymphocytic leukemia (B-CLL) in a dose- and time-dependent manner.^53^ The resistance to Akt/mTOR inhibitors such as everolimus (RAD001) is a significant clinical problem in relapsed mantle cell lymphoma (MCL) patients. Fortunately, pretreatment with HCQ can efficiently overcome this resistance, resulting in the activation of the mitochondrial apoptotic pathway.^54^ These data illustrate a strategy of blocking activation of adaptive autophagy pathway to improve treatment outcomes in leukemia and MCL.

### Lung cancer

Epidermal growth factor receptor tyrosine kinase inhibitors (EGFR-TKIs) have been widely used in patients with non-small-cell lung cancer (NSCL). Unfortunately, the efficacy of these drugs is limited because of natural or acquired resistance. We report that autophagy can be activated by gefitinib or erlotinib in lung cancer cells, which contributes to the acquired drug resistance of EGFR-TKIs.^55^ Furthermore, the antimalaria drug CQ has been shown to enhance chemotherapy and radiation sensitivity in several preclinical models. CQ can not only potentiate the cytotoxicity of topotecan (TPT), but also substantially increase the effects of PI3K/mTOR inhibitor NVP-BEZ235 on induction of apoptosis, inhibition of colony formation and suppression of xenografts in nude mice.^56, 57^ A phase I study in advanced NSCL patients with prior clinical benefit from EGFR-TKIs suggests that HCQ with or without erlotinib is safe, and the recommended phase II dose for HCQ with erlotinib 150 mg is 1000 mg daily.^58^ Although no difference in survival was elaborated, this study explored the safety of adding HCQ to erlotinib. To assess the efficacy of the combination of HCQ with conventional chemotherapeutics, several clinical trials have been launched.

### Pancreatic adenocarcinoma

The cytoprotective role of autophagy in response to chemotherapy has been confirmed in the treatment of pancreatic cancer cells.^59^ In agreement with this, Mirzoeva *et al.*^60^ showed that autophagy suppression with CQ promotes antitumor activity of PI3K/mTOR inhibitor for the treatment of pancreatic adenocarcinoma (PDAC) *in vitro* and *in vivo*. In addition to CQ, the efficacy and side effects of adding HCQ to conventional therapy are being investigated through several clinical trials.

### Prostate cancer and renal cell carcinoma

Several recent studies have indicated that autophagy functions as a survival mechanism to promote chemoresistance in prostate and renal cancer cells.^61, 62^ Autophagy activation protects against ER stress-induced cell death, whereas inhibition of autophagy significantly suppresses PC-3 prostate tumor growth *in vivo*.^63^ Moreover, inhibition of autophagy by either HCQ or Beclin-1/Atg5 small interfering RNA enhances ABT-737 cytotoxicity and ursolic acid-induced apoptosis in prostate cancer cells.^64, 65^ Administration of high-dose interleukin-2 (HDIL-2) has durable complete and partial responses in patients with metastatic renal cell carcinoma. However, HDIL-2 treatment is often limited by side effects, because of a cytokine-induced systemic autophagic syndrome. Liang *et al.*^66^ found that the combination of IL-2 with CQ increases antitumor effects and decreases toxicity when compared with IL-2 treatment alone, which provide a novel clinical strategy to enhance the efficacy of HDIL-2 immunotherapy for patients with renal cell carcinoma. So far, two active trials (NCT 01144169 and NCT 01550367) are investigating the efficacy of adding HCQ to IL-2.

### Ovarian cancer

Ovarian cancer has poor prognosis and is frequently resistant to chemotherapy. In this issue, it has been presented that autophagy may be a factor in drug resistance and poor survival in clear cell ovarian cancer patients.^67^ Nucleus accumbens-1 (NAC1) can mediate resistance to cisplatin in ovarian cancer cell lines because of activation of autophagy.^68^ FTY720, a sphingosine analog, may enhance autophagic flux when treated as a new chemotherapeutic agent for ovarian cancer, and blockade of autophagy aggravates necrotic ovarian cancer cell death in response to FTY720.^69^ Now, the effect of combining HCQ with sorafenib is being assessed in FIGO stage III or stage IV ovarian cancer, or extraovarian peritoneal carcinoma, or fallopian tube carcinoma failing or ineligible for first-line therapy.

## Other Compounds and Strategies

In addition to antimalarial drugs, inhibition of autophagy by either pharmacological approaches or via genetic silencing of autophagy regulatory genes such as *Beclin 1*, *ATG6*, *ATG5*, *ATG7* or *ATG12* (Table 3) also results in sensitization to a variety of therapeutic agents. Different autophagy inhibitors block the autophagic process at different stages. For example, antimalarial drugs or bafilomycin A1 can inhibit autophagosome fusion with lysosomes and autophagosome degradation in the final stage of autophagy. Class III PI3K inhibitors (3-methyladenine (3-MA), LY294002 and Wortmannin) or knockdown of autophagy regulatory genes are involved in the initiation/expansion stage of autophagy.^70, 71, 72, 73^ So far, different autophagy inhibitors or genetic knockout of autophagy regulatory genes have been developed and used in the study of autophagy in cancer chemotherapy.^74, 75, 76, 77, 78^

## The Molecular Mechanisms of Protective Autophagy-Mediated Chemoresistance

**Figure 4:**
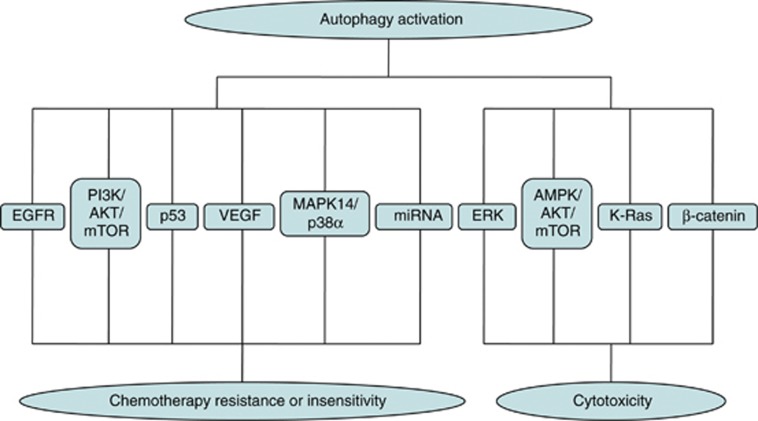
The molecular mechanisms of autophagy activation in response to chemotherapeutic agents. The activation of autophagy either leads to cancer cell chemoresistance via EGFR signaling, PI3K/AKT/ mTOR pathways, p53, VEGF, MAPK14/p38*α* signaling and microRNA or potentiates autophagic cell death through AMPK/AKT1/mTOR axis, which depends on the tumor types and treatment characteristic

Although many anticancer therapeutic strategies can induce autophagic cell death, a majority of pertinent studies have been conducted to indicate that autophagy is a protective mechanism associated with increased resistance to chemotherapy. Induction of autophagy has emerged as a drug resistance mechanism that promotes cancer cell survival. There are a number of different mechanisms through which the autophagy-related functions of promoting the survival of tumor cells under the treatment of anticancer drugs (Figure 4).

### EGFR signaling

Epidermal growth factor is a key regulatory factor for cell survival. Through its binding to cell surface receptors, EGF can induce the activation of three signaling pathways which are important to the initiation and progression of cancers, Ras/MAPK, PI3K/Akt and JAK/STATs.^79^ In the previous study, we confirmed biochemically and morphologically that autophagy can be activated by gefitinib or erlotinib, which was accompanied by the inhibition of the PI3K/Akt/mTOR signaling pathway. Furthermore, blockage of autophagy by the pharmacological inhibitors or gene silence greatly enhanced cytotoxicity of gefitinib or erlotinib.^55^ PD168393, an EGFR-TKI, may induce autophagy as a cytostatic but not a cytotoxic response in malignant peripheral nerve sheath tumor (MPNST) cells that was accompanied by suppression of Akt and mTOR activation; moreover, suppression of autophagy by CQ increased caspase activation.^80^ These results indicate that EGFR-TKIs can induce autophagy to promote tumor cell survival in response to targeted chemotherapies, and suppression of autophagy can augment the growth inhibitory effect of these drugs through inhibition of the PI3K/Akt/mTOR signaling pathway.

### PI3K/AKT/mTOR pathways

In addition to EGFR, the aberrant expression of PI3K/AKT/mTOR is also known to be a key regulator of authophagy. Genetic and pharmacologic autophagy blockade via PI3K/mTOR inhibition may reverse apoptotic resistance and result in significant cell apoptosis.^81^ NVP-BEZ235 (BEZ235) is a novel, orally bioavailable dual PI3K/mTOR inhibitor that has exerted a positive effect on autophagy. A recent study suggests that NVP-BEZ235 could induce apoptosis and autophagy; moreover, the combination treatment of NVP with autophagy inhibitors lead to enhanced RCC cell apoptosis.^62, 82^ Benzyl isothiocyanate (BITC), a dietary chemopreventive agent, is also found to induce protective autophagy in human prostate cancer cells via inhibition of mTOR signaling pathway, and inhibition of autophagy using 3-MA increased BITC-induced apoptosis.^83^ Taken together, targeting PI3K/AKT/mTOR-autophagy pathways displays a well-recognized contribution to overcome chemotherapy resistance and sensitize the tumor cells to anticancer therapy.

### p53, VEGF and MAPK14/p38*α* signaling

As a well-known tumor-suppressor gene, p53 is also involved in autophagic regulation. In a mouse model of c-Myc-driven lymphoma, inhibition of autophagy with either CQ or ATG5 shRNA promotes tumor cell death by p53 activation.^84^ Stanton *et al.*^85^ show that the VEGF-C/NRP-2 axis is involved in the activation of autophagy, and VEGF-C or NRP-2 depletion contributes to cytotoxic drug-mediated cell death. In addition, the recent studies have provided strong evidence that the MAPK14/p38 signaling is involved in cancer cell resistance to chemotherapy treatment. Paillas *et al.*^39^ investigated the relationship between MAPK14/p38, autophagy and resistance to irinotecan and found that MAPK14/p38 was activated and triggered survival-promoting autophagy to protect tumor cells against the cytotoxic effects of irinotecan. Furthermore, p38MAPK activation is considered a key determinant in the cellular response to 5-FU by controlling the balance between apoptosis and autophagy.^40^

### MicroRNAs

MicroRNAs (miRNAs) are a class of small, noncoding, endogenously encoded, single-stranded RNAs that regulate gene expression at the post-transcriptional level. Recently, a number of miRNAs have been reported to be deeply involved in resistance or sensitization to chemotherapy.^86, 87^ miR-30a, a member of miR-30 family, is a potent inhibitor of autophagy by selectively downregulating Beclin 1 and Atg5 expression. Targeting miR-30a promotes autophagy in response to imatinib treatment and enhances imatinib resistance against CML including primary stem and progenitor cells.^88, 89^ Moreover, the blockade of autophagy by miR-30a expression or 3-MA significantly increased *cis*-DDP-induced apoptosis in cancer cells.^90^ Downregulation of miR-199a-5p is observed in most HCC tissues of patients. More importantly, downregulation of miR-199a-5p increases cisplatin resistance by activating autophagy in HCC cells.^91^ Cisplatin-induced ATM-dependent phosphorylated (p)-ΔNp63*α* also plays an important role in chemoresistance. Further research shows that the p-ΔNp63*α*-induced miR-885-3p might contribute to regulation of apoptosis and/or autophagy in squamous cell carcinoma cells upon cisplatin exposure.^92^ Although the relationship between miRNA, autophagy and anticancer therapy resistance is quite complicated, and has not been well elucidated, miRNA may underlie key aspects of chemotherapy resistance.

## Chloroquine and Its Derivative: Just Act as Autophagy Inhibitors?

CQ and its derivative are currently the only autophagy inhibitors available for clinical treatment of patients. In ongoing cancer treatment clinical trials, HCQ is often used in combination with chemotherapeutic drugs, radiotherapy, some targeted therapies and immunotherapy. The treatment of CQ or HCQ can inhibit autophagy-related survival function and exert their anticancer action. However, emerging data indicate that the ability of CQ and its derivative to inhibit the final degradative step of autophagy may not be the only mechanism by which they exert anticancer action; CQ and HCQ may also affect other pathways such as lysosomal membrane permeabilization.

Recently, Maycotte *et al.*^36^ reported that CQ could sensitize breast cancer cells to chemotherapy independent of autophagy inhibition, as sensitization was not mimicked by Atg12, Beclin 1 knockdown or bafilomycin A1 treatment, and occurred even in the absence of Atg12. In further studies, CQ and HCQ are shown to function as lysosomotropic agents to promote lysosomal membrane permeabilization (LMP), resulting in signs of apoptosis. The research from Enzenmüller *et al.*^93^ shows that PI3K/mTOR inhibitor PI103 enhances the lysosomal compartment by increasing its volume and function, whereas CQ destabilizes lysosomal membranes. Together, CQ overcomes resistance of lung carcinoma cells to PI103-induced apoptosis by cooperating with PI103 to trigger lysosome-mediated apoptosis. An additional report suggests that induction of lysosome-mediated apoptosis rather than inhibition of autophagy is critical for the CQ-mediated sensitization to BEZ235-induced apoptosis, as lysosomal enzyme inhibitors significantly decrease BEZ235- and CQ-induced drop of mitochondrial membrane potential (MMP) and caspase-dependent apoptosis.^94^ Similar to the potential of CQ, HCQ can also induce LMP and Bax/Bak-dependent MMP to trigger caspase activation.^95^

Taken together, these data indicate that the success of clinical trials using CQ or HCQ combined with other anticancer agents might not be due to CQ or HCQ effects on autophagy induced by chemotherapeutic drugs, as the effect may be mediated by mechanisms other than its inhibition of autophagy. Thus, a better knowledge of the molecular mechanisms and cellular targets of CQ or HCQ should be considered in the ongoing clinical trials where CQ or HCQ are used as autophagy inhibitors.

## Autophagy-Mediated Cell Death Mechanism Contributes to Efficacy of Anticancer Drugs

Despite its a clear prosurvival role, autophagy has also been viewed as having a prodeath role under certain circumstances and following treatment with a specific set of chemotherapeutic agents, either by enhancing the induction of apoptosis or mediating ‘autophagic cell death'.

Increasing evidence supports that autophagy may mediate cell death in cancer cells which are apoptosis defective or hard to induce. Xiong *et al.*^96^ found that autophagic cell death could be induced in PUMA- or Bax-deficient human colon cancer cells after the treatment with 5-FU, consequently followed by decreased cancer proliferation. Suberoylanilide hydroxamic acid (SAHA), a prototype of the newly developed HDAC inhibitor, induces autophagic cell death in tamoxifen-resistant MCF-7 breast cancer cells and significantly reduces the tumor growth *in vitro* and *in vivo*.^97^ NVP-BEZ235 is demonstrated to inhibit cisplatin-resistant urothelial cancer cell proliferation by activating autophagic flux and cell cycle arrest, but not inducing apoptotic cell death.^98^ The antidepressants maprotiline and fluoxetine induce autophagic cell death in drug-resistant Burkitt's lymphoma (BL), which supports a new mechanistic role for maprotiline and fluoxetine as novel proautophagic agents in the treatment of resistant BL.^99^ These data indicate that autophagic cell death can be induced as an alternative cell death mechanism when cells fail to undergo apoptosis.

In addition, induction of autophagic cell death is also an alternative approach to kill tumor cells without resistance to anticancer drugs. Ursolic acid promotes cancer cell death by inducing Atg5-dependent autophagy.^100^ FK-16, derived from the anticancer peptide LL-37, induces caspase-independent apoptosis and autophagic cell death in colon cancer cells.^101^ The mTOR inhibitor RAD001 potentiates autophagic cell death induced by temozolomide in a GBM cell line.^102^ Novel monofunctional platinum (II) complex Mono-Pt induces apoptosis-independent autophagic cell death in human ovarian carcinoma cells, distinct from cisplatin.^103^ Sorafenib and SC-59, which is a novel sorafenib derivative, induce autophagic cell death in hepatocellular carcinoma cells in a dose- and time-dependent manner.^104^ Recently, cannabinoids have been shown to exert their anticancer activity in glioma, pancreatic cancer and hepatocellular carcinoma via stimulation of autophagy-mediated cell death.^105, 106, 107, 108^ Moreover, the combination of cannabinoids and TMZ strongly activates autophagy-mediated cancer cell death, resulting in a strong antitumoral action in both TMZ-sensitive and TMZ-resistant tumors.^109^ These studies present new insights into our understanding of the relationship between autophagy and anticancer efficacy and provide a potential therapeutic strategy for the management of some of these tumors.

Several genes and signal pathways contribute to autophagic cell death in cancer cells (Figure 4). The AMPK/AKT1/mTOR axis is critical for regulation of autophagic cell death. Tanshinone IIA induces autophagic cell death via activation of AMPK/ERK and inhibition of mTOR and p70 S6K in KBM-5 leukemia cells.^110^ Cannabinoids can trigger autophagic cell death in an ER stress and Akt/mTORC1-dependent manner.^105, 106, 107, 108^ 4-Hydroxytamoxifen induces autophagic death through K-Ras degradation.^111^ Diarylquinoline compounds induce a autophagic cell death by inhibiting the Akt pathway and increasing reactive oxygen species in human nasopharyngeal carcinoma cells.^112^ *β*-Catenin is also involved in activation of autophagic cell death.^113^ These results further strengthen the connection between autophagy and anticancer efficacy.

Recently, autophagy has been shown to precede apoptosis or act in parallel with this cellular process in addition to being an alternative mechanism to cell death when apoptosis is inhibited. It has been acknowledged that autophagy precedes caspase-dependent apoptosis.^114, 115^ Several studies demonstrate that autophagy precedes apoptosis and acts as a protective mechanism in cancer cells.^116, 117, 118^ Therefore, the autophagy induction may exert other possibilities, which should be considered in the design of new treatments for these malignancies.

## Conclusions and Perspectives

Autophagy is a lysosomal degradation process usually activated in response to adverse microenvironmental stresses. Autophagy itself fulfils a dual role, having tumor-promoting and tumor-suppressing properties. As a response to anticancer treatments, whether autophagy activation leads to cell survival or cell death remains controversial. It is consensus that the outcomes of autophagy activation are highly depended on the tumor types and treatment characteristic.^119, 120^

Resistance to chemotherapy is a major obstacle for the success of cancer therapy. Although the controversy about the prosurvival or anticancer effect of autophagy is still heated, the data *in vitro* and *in vivo* seem more to support the idea that autophagy facilitates the cancer cells' resistance to chemotherapy treatment, and inhibition of autophagy may potentiate the resensitization of therapeutic-resistant cancer cells to the anticancer drugs.^26, 121^ Several autophagy inhibitors such as CQ and its derivative HCQ have been studied in preclinical models. Although CQ or HCQ augments cytotoxicity in combination with several anticancer drugs, autophagy researchers should be careful when interpreting experiments in which CQ or HCQ treatment is used, as the effect may be mediated by mechanisms other than its inhibition of autophagy.

Currently, the combination of autophagy inhibitors with cytotoxic drugs is attracting more and more attention in cancer therapy. However, the study of the ability of autophagy inhibitors to overcome resistance to anticancer therapies and how this relates to the regulation of tumor microenvironmental stresses raises many issues. First, the question of whether we should try to enhance or inhibit autophagy in cancer treatment is not straightforward as it might vary according to cell type, the stress signal and other circumstances. What is needed to be better experimentally addressed include elucidating the impact of the tumor microenvironment on autophagy function, determining the role(s) of autophagy in the regulation of therapeutic sensitivity and defining novel mechanism by which autophagy inhibition can overcome chemotherapy resistance and sensitize the tumor cells to anticancer therapy. Second, to maximize the potential to be applied for more stringent clinical study, new and reliable methods for measuring autophagy in clinical samples need to be developed. Third, the ocular toxicities and minimal single-agent anticancer efficacy of CQ or HCQ have restricted its clinical application. New and exciting autophagy inhibitors are worthy of further investigation in the future. Thus, establishment of more effective and safe combinatorial therapeutic strategies using autophagy inhibitors will be necessary in the future. However, our increased understanding of the functional relevance of autophagy within the tumor microenvironment and ongoing dialogue between emerging laboratory and clinical research of targeting autophagy will hopefully provide a promising therapeutic strategy to circumvent resistance and enhance the effects of anticancer therapies for cancer patients.

## Figures and Tables

**Table 1:** Autophagy in response to chemotherapy in different types of cancers

**Class**	**Target**	**Type of cancer**	**Autophagy role**	**Method used to evaluate autophagy**	**References**
*Autophagy inducers*					
Aurora kinase A	mTOR	Breast	Prosurvival	siRNA (LC3, Atg5) CQ Bafilomycin A	^27^
Suberoylanilide hydroxamic acid (SAHA)	HDAC inhibitor	CML	Prosurvival	3-MA Bafilomycin A	^29^
		Breast	Prodeath	3-MA	^97^
Epirubicin (EPI)	Anthracyclines	Breast	Prosurvival	siRNA (Beclin 1, Atg7) Bafilomycin A	^34^
5-Fluorouracil	Thymidylate synthase inhibitor	Colorectal	Prosurvival	siRNA (Atg7) 3-MA	^37, 40, 41, 42^
			Prodeath	3-MA	^96^
Atorvastatin	AMPK	Digestive malignancies	Prosurvival	siRNA (Atg5) Bafilomycin A	^38^
Irinotecan	MAPK14/p38*α*	Colorectal	Prosurvival	siRNA (Atg5, Atg7) Bafilomycin A 3-MA	^39^
Cisplatin	Genotoxic stress	Esophageal	Prosurvival	3-MA	^43^
Oxaliplatin	Genotoxic stress	Hepatocellular carcinoma	Prosurvival	siRNA (Atg5) CQ 3-MA	^48^
Bevacizumab	Angiogenesis inhibitor	Hepatocellular carcinoma	Prosurvival	CQ 3-MA	^49^
Sorafenib	ER stress	Hepatocellular carcinoma	Prosurvival	CQ	^50^
	Genotoxic stress		Prodeath	siRNA (Beclin 1)	^104^
High-mobility group box 1 protein (HMGB1)	DAMP molecule	CML	Prosurvival		^52^
Gefitinib or Erlotinib	EGFR tyrosine kinase inhibitor	Lung	Prosurvival	siRNA (Atg5, Atg7) CQ 3-MA	^55, 58^
Topotecan	Genotoxic stress	Lung	Prosurvival	CQ	^56^
RAGE	Genotoxic or metabolic stress	Pancreatic	Prosurvival		^59^
NVP-BEZ235	PI3K/AKT/mTOR inhibitor	Renal	Prosurvival		^62^
		Urothelial	Prodeath	CQ	^98^
Ursolic acid	Genotoxic stress	Prostate	Prosurvival	siRNA (Atg5, Beclin 1) 3-MA	^64^
		Cervical	Prodeath	siRNA (Atg5) Wortmannin	^100^
Imatinib	Tyrosine kinase inhibitor	Glioma	Prosurvival	Bafilomycin A RTA 203	^71^
			Prodeath	siRNA (Atg5, Beclin 1) 3-MA	^71^
FK-16	Fragment of LL-37	Colon	Prodeath	siRNA (Bax, Bcl-2)	^101^
Temozolomide	Genotoxic stress	Glioblastoma	Prodeath	mTOR inhibitor RAD001	^102^
Mono-Pt	Genotoxic stress	Ovarian	Prodeath	siRNA (Atg7, Beclin 1) 3-MA CQ Bafilomycin A	^103^
Cannabinoids	ER stress	Glioma	Prodeath	siRNA (Atg1)	^105^
	AMPK	Pancreatic	Prodeath	3-MA CQ	^106^
		Hepatocellular carcinoma	Prodeath	siRNA (Atg5) 3-MA	^108^
*Autophagy inhibitors*					
CQ	Lysosomotropic agent	Breast	Prosurvival		^35^
HCQ		Esophageal	Prosurvival		^43, 44, 45^
		Hepatocellular carcinoma	Prosurvival		^50^
		Lung	Prosurvival		^56, 57^
		Pancreatic	Prosurvival		^60^

**Table 2:** Active clinical trials combining the autophagy inhibitor HCQ with anticancer therapies

**Identifier**	**Cancer type**	**Drugs**	**Phase**	**Title**
NCT00969306	NSCLC	CQ+cisplatin Etoposide	I/II	Cisplatin, etoposide and escalating CQ in extensive disease SCLC
NCT00809237	NSCLC	HCQ+gefitinib	I/II	Hydroxychloroquine and gefitinib to treat lung cancer
NCT01649947	NSCLC	HCQ+paclitaxel and carboplatin	II	Modulation of autophagy in patients with advanced/recurrent non-small-cell lung cancer – phase II
NCT00977470	Advanced NSCLC and (EGFR) mutations	HCQ+erlotinib	II	Erlotinib with or without hydroxychloroquine in chemonaive advanced NSCLC and (EGFR) mutations
NCT00933803	Advanced or recurrent NSCLC	HCQ+carboplatin, paclitaxel, bevacizuma		Carboplatin, paclitaxel, bevacizumab and HCQ in advanced or recurrent NSCLC
NCT01292408	Breast cancer	HCQ	II	Autophagy inhibition using hydroxychloroquine in breast cancer patients
NCT00765765	Breast cancer	HCQ+ixabepilone	I/II	Ixabepilone and HCQ in metastatic breast cancer
NCT01023477	DCIS	CQ+tamoxifen	I/II	Neoadjuvant tamoxifen, tamoxifen+CQ, or CQ in DCIS
NCT01510119	Renal cell carcinoma	HCQ and RAD001(p.o. 10 mg/day)	I/II	Autophagy inhibition to augment mTOR inhibition: a phase I/II trial of RAD001 and hydroxychloroquine in patients with previously treated renal cell carcinoma
NCT01144169	Renal cell carcinoma	HCQ+high dose interleukin-2 and other systemic therapies	I	Study of hydroxychloroquine before surgery in patients with primary renal cell carcinoma
NCT01550367	Renal cell carcinoma	HCQ+IL-2	I/II	Study of hydroxychloroquine and aldesleukin in renal cell carcinoma patients (RCC)
NCT00726596	Prostate cancer	HCQ	II	Hydroxychloroquine in treating patients with rising PSA levels after local therapy for prostate cancer
NCT01128296	Pancreatic cancer	HCQ+gemcitabine	I/II	Study of presurgery gemcitabine+hydroxychloroquine (GcHc) in stage IIb or III adenocarcinoma of the pancreas
NCT01273805	Pancreatic cancer	HCQ	II	Hydroxychloroquine in previously treated patients with metastatic pancreatic cancer
NCT01506973	Pancreatic cancer	HCQ+gemcitabine/abraxane	I/II	A phase I/II/pharmacodynamic study of hydroxychloroquine in combination with gemcitabine/abraxane to inhibit autophagy in pancreatic cancer
NCT01128296	Pancreatic cancer	HCQ+gemcitabine	I/II	Study of Pre-surgery Gemcitabine+hydroxychloroquine (GcHc) in stage IIb or III adenocarcinoma of the pancreas
NCT01494155	Pancreatic cancer	HCQ+capecitabine+photon radiation	II	Short-course radiation therapy with proton beam capecitabine and hydroxychloroquine for resectable pancreatic cancer
NCT01206530	Colorectal cancer	HCQ+FOLFOX/bevacizumab	I/II	FOLFOX/Bevacizumab/Hydroxychloroquine (HCQ) in colorectal cancer
NCT01006369	Metastatic colorectal cancer	HCQ+capecitabine, oxaliplatin, and bevacizumab	II	Hydroxychloroquine, capecitabine, oxaliplatin, and bevacizumab in treating patients with metastatic colorectal cancer
NCT00224978	Glioblastoma	CQ	III	Adjuvant CQ *versus* placebo in glioblastoma
NCT00486603	Glioblastoma	HCQ+temozolomide	I/II	Adjuvant radiation, temozolomide and HCQ in newly resected GBM
NCT00962845	Melanoma	HCQ	No phase specified	Hydroxychloroquine in patients with stage III or Stage IV melanoma that can be removed by surgery
NCT00568880	Multiple myeloma	HCQ+bortezomib	I/II	Hydroxychloroquine and bortezomib in treating patients with relapsed or refractory multiple myeloma
NCT01480154	Advanced solid tumors or prostate or renal cancer	HCQ+MTD of Akt inhibitor MK2206 (MK-2206)	I	Phase I study of Akt inhibitor MK2206 and hydroxychloroquine in patients with advanced solid tumors or prostate or renal cancer
NCT00909831	Metastatic solid tumors	HCQ+temsirolimus	I	Hydroxychloroquine and temsirolimus in treating patients with metastatic solid tumors that have not responded to treatment
NCT00813423	Advanced solid tumors	HCQ+sunitinib	I	Sunitinib and Hydroxychloroquine in treating patients with advanced solid tumors that have not responded to chemotherapy
NCT01023737	Advanced solid tumors	HCQ+vorinostat	I	Vorinostat and HCQ in advanced solid tumors
NCT01417403	Solid tumors undergoing radiation therapy for bone metastases	HCQ	I	Hydroxychloroquine in treating patients with solid tumors undergoing radiation therapy for bone metastases
NCT01266057	Advanced cancer	HCQ+the highest tolerable dose of sirolimus or vorinostat	I	Sirolimus or vorinostat and hydroxychloroquine in advanced cancer
NCT00714181	Metastatic or unresectable solid tumors	HCQ+temozolomide	I	Hydroxychloroquine and temozolomide in treating patients with metastatic or unresectable solid tumors
NCT01227135	CML	HCQ+imatinib	II	Imatinib mesylate with or without hydroxychloroquine in treating patients with chronic myeloid leukemia
NCT01634893	Ovarian cancer	HCQ+sorafenib	I	Oral hydroxychloroquine plus oral sorafenib to treat epithelial ovarian cancer FIGO stage III or stage IV, or extraovarian peritoneal carcinoma, or fallopian tube carcinoma failing or ineligible for first-line therapy

NSCLC, non-small-cell lung cancer; CML, chronic myeloid leukemia; EGFR, epidermal growth factor receptor; MTD, maximum tolerated dose; HCQ, hydroxychloroquine

**Table 3:** The strategies for autophagy inhibition

**Strategies**	**Target**	**The effect on autophagy**
*Pharmacological approaches*		
Chloroquine	Lysosomal pH	Inhibit autophagosome fusion with lysosomes and autophagosome degradation
Hydroxychloroquine	Lysosomal pH	Inhibit autophagosome fusion with lysosomes and autophagosome degradation
Monensin	Change endocytic and lysosomal pH	Inhibit the initiation/expansion stage of autophagy
Bafilomycin A 1	Class III PI3K inhibitor	Inhibit the initiation/expansion stage of autophagy
3-Methyladenine	Class III PI3K inhibitor	Inhibit the initiation/expansion stage of autophagy
Wortmannin	Class III PI3K inhibitor	Inhibit the initiation/expansion stage of autophagy
LY294002	Class III PI3K inhibitor	Inhibit the initiation/expansion stage of autophagy
Pyrvinium	Class III PI3K inhibitor	Inhibit the initiation/expansion stage of autophagy
Genetic silencing of autophagy regulatory genes		Inhibit the initiation/expansion stage of autophagy

## Abbreviations

PI3K: phosphatidylinositol 3-kinase
mTOR: mammalian target of rapamycin
AMPK: AMP-activated protein kinase
ER: endoplasmic reticulum
eIF2*α*: eukaryotic initiation factor 2*α*
HDAC: histone deacetylase
DDR: DNA damage response
CQ: chloroquine
HCQ: hydroxychloroquine
EPI: epirubicin
5-FU: 5-fluorouracil
MAPK: mitogen-activated protein kinase
GBM: glioblastoma
TMZ: temozolomide
MTD: maximum tolerated dose
HCC: hepatocellular carcinoma
MCL: mantle cell lymphoma
DAMP: damage-associated molecular pattern
B-CLL: B-chronic lymphocytic leukemia
EGF: epidermal growth factor
EGFR-TKI: epidermal growth factor receptor tyrosine kinase inhibitor
TPT: topotecan
PDAC: pancreatic adenocarcinoma
HDIL-2: high-dose interleukin-2
NAC1: nucleus accumbens-1
3-MA: 3-methyladenine
MPNST: malignant peripheral nerve sheath tumor
BITC: benzyl isothiocyanate
LMP: lysosomal membrane permeabilization
MMP: mitochondrial membrane potential
SAHA: suberoylanilide hydroxamic acid
BL: Burkitt's lymphoma

## References

[bib1] Ringborg U Platz A Chemotherapy resistance mechanisms Acta Oncol 1996 35 76 80 9142973 10.3109/02841869609083976

[bib2] Szakács G Paterson JK Ludwig JA Booth-Genthe C Gottesman MM Targeting multidrug resistance in cancer Nat Rev Drug Discov 2006 5 219 234 16518375 10.1038/nrd1984

[bib3] Yang Z Klionsky DJ Eaten alive: a history of macroautophagy Nat Cell Biol 2010 12 814 822 20811353 10.1038/ncb0910-814 PMC3616322

[bib4] Klionsky DJ Abdalla FC Abeliovich H Abraham RT Acevedo-Arozena A Adeli K Guidelines for the use and interpretation of assays for monitoring autophagy Autophagy 2012 8 445 544 22966490 10.4161/auto.19496 PMC3404883

[bib5] Sui X Jin L Huang X Geng S He C Hu X p53 signaling and autophagy in cancer: a revolutionary strategy could be developed for cancer treatment Autophagy 2011 7 565 571 21099252 10.4161/auto.7.6.14073

[bib6] Yang Z Klionsky DJ An overview of the molecular mechanism of autophagy Curr Top Microbiol Immunol 2009 335 1 32 19802558 10.1007/978-3-642-00302-8_1 PMC2832191

[bib7] Kondo Y Kanzawa T Sawaya R Kondo S The role of autophagy in cancer development and response to therapy Nat Rev Cancer 2005 5 726 734 16148885 10.1038/nrc1692

[bib8] Maycotte P Thorburn A Autophagy and cancer therapy Cancer Biol Ther 2011 11 127 137 21178393 10.4161/cbt.11.2.14627 PMC3047083

[bib9] Janku F McConkey DJ Hong DS Kurzrock R Autophagy as a target for anticancer therapy Nat Rev Clin Oncol 2011 8 528 539 21587219 10.1038/nrclinonc.2011.71

[bib10] Glick D Barth S Macleod KF Autophagy: cellular and molecular mechanisms J Pathol 2010 221 3 12 20225336 10.1002/path.2697 PMC2990190

[bib11] Cecconi F Levine B The role of autophagy in mammalian development: cell makeover rather than cell death Dev Cell 2008 15 344 357 18804433 10.1016/j.devcel.2008.08.012 PMC2688784

[bib12] Din FV Valanciute A Houde VP Zibrova D Green KA Sakamoto K Aspirin inhibits mTOR signaling, activates AMP-activated protein kinase, and induces autophagy in colorectal cancer cells Gastroenterology 2012 142 1504 1515 22406476 10.1053/j.gastro.2012.02.050 PMC3682211

[bib13] Yu L McPhee CK Zheng L Mardones GA Rong Y Peng J Termination of autophagy and reformation of lysosomes regulated by mTOR Nature 2010 465 942 946 20526321 10.1038/nature09076 PMC2920749

[bib14] He C Klionsky DJ Regulation mechanisms and signaling pathways of autophagy Annu Rev Genet 2009 43 67 93 19653858 10.1146/annurev-genet-102808-114910 PMC2831538

[bib15] Yang Z Klionsky DJ Mammalian autophagy: core molecular machinery and signaling regulation Curr Opin Cell Biol 2010 22 124 131 20034776 10.1016/j.ceb.2009.11.014 PMC2854249

[bib16] Tsuchihara K Fujii S Esumi H Autophagy and cancer: dynamism of the metabolism of tumor cells and tissues Cancer Lett 2009 278 130 138 19004545 10.1016/j.canlet.2008.09.040

[bib17] Hardie DG AMPK and Raptor: matching cell growth to energy supply Mol Cell 2008 30 263 265 18471972 10.1016/j.molcel.2008.04.012

[bib18] Mahoney E Lucas DM Gupta SV Wagner AJ Herman SE Smith LL ER stress and autophagy: new discoveries in the mechanism of action and drug resistance of the cyclin-dependent kinase inhibitor flavopiridol Blood 2012 120 1262 1273 22740450 10.1182/blood-2011-12-400184 PMC3418721

[bib19] Kouroku Y Fujita E Tanida I Ueno T Isoai A Kumagai H ER stress (PERK/eIF2alpha phosphorylation) mediates the polyglutamine-induced LC3 conversion, an essential step for autophagy formation Cell Death Differ 2007 14 230 239 16794605 10.1038/sj.cdd.4401984

[bib20] Park KJ Lee SH Lee CH Jang JY Chung J Kwon MH Upregulation of Beclin-1 expression and phosphorylation of Bcl-2 and p53 are involved in the JNK-mediated autophagic cell death Biochem Biophys Res Commun 2009 382 726 729 19318089 10.1016/j.bbrc.2009.03.095

[bib21] Kim E Goraksha-Hicks P Li L Neufeld TP Guan KL Regulation of TORC1 by Rag GTPases in nutrient response Nat Cell Biol 2008 10 935 945 18604198 10.1038/ncb1753 PMC2711503

[bib22] Ciuffreda L Di Sanza C Incani UC Milella M The mTOR pathway: a new target in cancer therapy Curr Cancer Drug Targets 2010 10 484 495 20384580 10.2174/156800910791517172

[bib23] Botrugno OA Robert T Vanoli F Foiani M Minucci S Molecular pathways: old drugs define new pathways: non-histone acetylation at the crossroads of the DNA damage response and autophagy Clin Cancer Res 2012 18 2436 2442 22512979 10.1158/1078-0432.CCR-11-0767

[bib24] Shubassi G Robert T Vanoli F Minucci S Foiani M Acetylation: a novel link between double-strand break repair and autophagy Cancer Res 2012 72 1332 1335 22422989 10.1158/0008-5472.CAN-11-3172

[bib25] Zhao Y Chen H Shang Z Jiao B Yuan B Sun W SD118-xanthocillin X (1), a novel marine agent extracted from Penicillium commune, induces autophagy through the inhibition of the MEK/ERK pathway Mar Drugs 2012 10 1345 1359 22822377 10.3390/md10061345 PMC3397444

[bib26] Hu YL Jahangiri A Delay M Aghi MK Tumor cell autophagy as an adaptive response mediating resistance to treatments such as antiangiogenic therapy Cancer Res 2012 72 4294 4299 22915758 10.1158/0008-5472.CAN-12-1076 PMC3432684

[bib27] Zou Z Yuan Z Zhang Q Long Z Chen J Tang Z Aurora kinase A inhibition-induced autophagy triggers drug resistance in breast cancer cells Autophagy 2012 8 1798 1810 23026799 10.4161/auto.22110 PMC3541289

[bib28] Firat E Weyerbrock A Gaedicke S Grosu AL Niedermann G Chloroquine or chloroquine-PI3K/Akt pathway inhibitor combinations strongly promote γ-irradiation-induced cell death in primary stem-like glioma cells PLoS One 2012 7 e47357 23091617 10.1371/journal.pone.0047357 PMC3473017

[bib29] Carew JS Nawrocki ST Kahue CN Zhang H Yang C Chung L Targeting autophagy augments the anticancer activity of the histone deacetylase inhibitor SAHA to overcome Bcr-Abl-mediated drug resistance Blood 2007 110 313 322 17363733 10.1182/blood-2006-10-050260 PMC1896119

[bib30] Sotelo J Briceno E Lopez-Gonzalez MA Adding chloroquine to conventional treatment for glioblastoma multiforme: a randomized, double-blind, placebo-controlled trial Ann Intern Med 2006 144 337 343 16520474 10.7326/0003-4819-144-5-200603070-00008

[bib31] Poole B Ohkuma S Effect of weak bases on the intralysosomal pH in mouse peritoneal macrophages J Cell Biol 1981 90 665 669 6169733 10.1083/jcb.90.3.665 PMC2111912

[bib32] Nilsson JR Does chloroquine, an antimalarial drug, affect autophagy in Tetrahymena pyriformis J Protozool 1992 39 9 16 1560421 10.1111/j.1550-7408.1992.tb01278.x

[bib33] Gunja N Roberts D McCoubrie D Lamberth P Jan A Simes DC Survival after massive hydroxychloroquine overdose Anaesth Intensive Care 2009 37 130 133 19157361 10.1177/0310057X0903700112

[bib34] Sun WL Chen J Wang YP Zheng H Autophagy protects breast cancer cells from epirubicin-induced apoptosis and facilitates epirubicin-resistance development Autophagy 2011 7 1035 1044 21646864 10.4161/auto.7.9.16521

[bib35] Schoenlein PV Periyasamy-Thandavan S Samaddar JS Jackson WH Barrett JT Autophagy facilitates the progression of ERalpha-positive breast cancer cells to antiestrogen resistance Autophagy 2009 5 400 403 19221464 10.4161/auto.5.3.7784

[bib36] Maycotte P Aryal S Cummings CT Thorburn J Morgan MJ Thorburn A Chloroquine sensitizes breast cancer cells to chemotherapy independent of autophagy Autophagy 2012 8 200 212 22252008 10.4161/auto.8.2.18554 PMC3336076

[bib37] Li J Hou N Faried A Tsutsumi S Kuwano H Inhibition of autophagy augments 5-fluorouracil chemotherapy in human colon cancer *in vitro* and *in vivo* model Eur J Cancer 2010 46 1900 1909 20231086 10.1016/j.ejca.2010.02.021

[bib38] Yang PM Liu YL Lin YC Shun CT Wu MS Chen CC Inhibition of autophagy enhances anticancer effects of atorvastatin in digestive malignancies Cancer Res 2010 70 7699 7709 20876807 10.1158/0008-5472.CAN-10-1626

[bib39] Paillas S Causse A Marzi L de Medina P Poirot M Denis V MAPK14/p38α confers irinotecan resistance to TP53-defective cells by inducing survival autophagy Autophagy 2012 8 1098 1112 22647487 10.4161/auto.20268 PMC3429546

[bib40] de la Cruz-Morcillo MA Valero ML Callejas-Valera JL Arias-González L Melgar-Rojas P Galán-Moya EM P38MAPK is a major determinant of the balance between apoptosis and autophagy triggered by 5-fluorouracil: implication in resistance Oncogene 2012 31 1073 1085 21841826 10.1038/onc.2011.321

[bib41] Sasaki K Tsuno NH Sunami E Tsurita G Kawai K Okaji Y Chloroquine potentiates the anti-cancer effect of 5-fluorouracil on colon cancer cells BMC Cancer 2010 10 370 20630104 10.1186/1471-2407-10-370 PMC2914703

[bib42] Sasaki K Tsuno NH Sunami E Kawai K Hongo K Hiyoshi M Resistance of colon cancer to 5-fluorouracil may be overcome by combination with chloroquine, an *in vivo* study Anticancer Drugs 2012 23 675 682 22561420 10.1097/CAD.0b013e328353f8c7

[bib43] Liu D Yang Y Liu Q Wang J Inhibition of autophagy by 3-MA potentiates cisplatin-induced apoptosis in esophageal squamous cell carcinoma cells Med Oncol 2011 28 105 111 20041317 10.1007/s12032-009-9397-3

[bib44] O'Donovan TR O'Sullivan GC McKenna SL Induction of autophagy by drug-resistant esophageal cancer cells promotes their survival and recovery following treatment with chemotherapeutics Autophagy 2011 7 509 524 21325880 10.4161/auto.7.6.15066 PMC3127212

[bib45] Chen YS Song HX Lu Y Li X Chen T Zhang Y Autophagy inhibition contributes to radiation sensitization of esophageal squamous carcinoma cells Dis Esophagus 2011 24 437 443 21166739 10.1111/j.1442-2050.2010.01156.x

[bib46] Hu YL DeLay M Jahangiri A Molinaro AM Rose SD Carbonell WS Hypoxia-induced autophagy promotes tumor cell survival and adaptation to antiangiogenic treatment in glioblastoma Cancer Res 2012 72 1773 1783 22447568 10.1158/0008-5472.CAN-11-3831 PMC3319869

[bib47] Rosenfeld MRGS Brem S Mikkelson T Wang D Piao S Davis L Pharmacokinetic analysis and pharmacodynamic evidence of autophagy inhibition in patients with newly diagnosed glioblastoma treated on a phase I trial of hydroxychloroquine in combination with adjuvant temozolomide and radiation (ABTC 0603) J Clin Oncol 2010 28 3086

[bib48] Ding ZB Hui B Shi YH Zhou J Peng YF Gu CY Autophagy activation in hepatocellular carcinoma contributes to the tolerance of oxaliplatin via reactive oxygen species modulation Clin Cancer Res 2011 17 6229 6238 21825039 10.1158/1078-0432.CCR-11-0816

[bib49] Guo XL Li D Sun K Wang J Liu Y Song JR Inhibition of autophagy enhances anticancer effects of bevacizumab in hepatocarcinoma J Mol Med (Berl) 2013 91 473 483 23052483 10.1007/s00109-012-0966-0 PMC3611041

[bib50] Shi YH Ding ZB Zhou J Hui B Shi GM Ke AW Targeting autophagy enhances sorafenib lethality for hepatocellular carcinoma via ER stress-related apoptosis Autophagy 2011 7 1159 1172 21691147 10.4161/auto.7.10.16818

[bib51] Liu L Yang M Kang R Wang Z Zhao Y Yu YX DAMP-mediated autophagy contributes to drug resistance Autophagy 2011 7 112 114 21068541 10.4161/auto.7.1.14005 PMC3039734

[bib52] Zhao M Yang M Yang L Yu Y Xie M Zhu S HMGB1 regulates autophagy through increasing transcriptional activities of JNK and ERK in human myeloid leukemia cells BMB Rep 2011 44 601 606 21944254 10.5483/bmbrep.2011.44.9.601

[bib53] Lagneaux L Delforge A Carlier S Massy M Bernier M Bron D Early induction of apoptosis in B-chronic lymphocytic leukaemia cells by hydroxychloroquine: activation of caspase-3 and no protection by survival factors Br J Haematol 2001 112 344 352 11167827 10.1046/j.1365-2141.2001.02553.x

[bib54] Rosich L Xargay-Torrent S López-Guerra M Campo E Colomer D Roué G Counteracting autophagy overcomes resistance to everolimus in mantle cell lymphoma Clin Cancer Res 2012 18 5278 5289 22879389 10.1158/1078-0432.CCR-12-0351

[bib55] Han W Pan H Chen Y Sun J Wang Y Li J EGFR tyrosine kinase inhibitors activate autophagy as a cytoprotective response in human lung cancer cells PLoS One 2011 6 e18691 21655094 10.1371/journal.pone.0018691 PMC3107207

[bib56] Wang Y Peng RQ Li DD Ding Y Wu XQ Zeng YX Chloroquine enhances the cytotoxicity of topotecan by inhibiting autophagy in lung cancer cells Chin J Cancer 2011 30 690 700 21959046 10.5732/cjc.011.10056 PMC4012269

[bib57] Xu CX Zhao L Yue P Fang G Tao H Owonikoko TK Augmentation of NVP-BEZ235's anticancer activity against human lung cancer cells by blockage of autophagy Cancer Biol Ther 2011 12 549 555 21738008 10.4161/cbt.12.6.16397 PMC3218593

[bib58] Goldberg SB Supko JG Neal JW Muzikansky A Digumarthy S Fidias P A phase I study of erlotinib and hydroxychloroquine in advanced non-small-cell lung cancer J Thorac Oncol 2012 7 1602 1608 22878749 10.1097/JTO.0b013e318262de4a PMC3791327

[bib59] Kang R Tang D Schapiro NE Livesey KM Farkas A Loughran P The receptor for advanced glycation end products (RAGE) sustains autophagy and limits apoptosis, promoting pancreatic tumor cell survival Cell Death Differ 2010 17 666 676 19834494 10.1038/cdd.2009.149 PMC3417122

[bib60] Mirzoeva OK Hann B Hom YK Debnath J Aftab D Shokat K Autophagy suppression promotes apoptotic cell death in response to inhibition of the PI3K-mTOR pathway in pancreatic adenocarcinoma J Mol Med (Berl) 2011 89 877 889 21678117 10.1007/s00109-011-0774-y

[bib61] Kaini RR Sillerud LO Zhaorigetu S Hu CA Autophagy regulates lipolysis and cell survival through lipid droplet degradation in androgen-sensitive prostate cancer cells Prostate 2012 72 1412 1422 22294520 10.1002/pros.22489 PMC3418419

[bib62] Li H Jin X Zhang Z Xing Y Kong X Inhibition of autophagy enhances apoptosis induced by the PI3K/AKT/mTor inhibitor NVP-BEZ235 in renal cell carcinoma cells Cell Biochem Funct 2013 31 427 433 23086777 10.1002/cbf.2917

[bib63] Kumano M Furukawa J Shiota M Zardan A Zhang F Beraldi E Cotargeting stress-activated Hsp27 and autophagy as a combinatorial strategy to amplify endoplasmic reticular stress in prostate cancer Mol Cancer Ther 2012 11 1661 1671 22675041 10.1158/1535-7163.MCT-12-0072 PMC4128402

[bib64] Shin SW Kim SY Park JW Autophagy inhibition enhances ursolic acid-induced apoptosis in PC3 cells Biochim Biophys Acta 2012 1823 451 457 22178132 10.1016/j.bbamcr.2011.10.014

[bib65] Saleem A Dvorzhinski D Santanam U Mathew R Bray K Stein M Effect of dual inhibition of apoptosis and autophagy in prostate cancer Prostate 2012 72 1374 1381 22241682 10.1002/pros.22487 PMC3840901

[bib66] Liang X De Vera ME Buchser WJ Romo de Vivar Chavez A Loughran P Beer Stolz D Inhibiting systemic autophagy during interleukin 2 immunotherapy promotes long-term tumor regression Cancer Res 2012 72 2791 2801 22472122 10.1158/0008-5472.CAN-12-0320 PMC3417121

[bib67] Gibson SB Autophagy in clear cell ovarian cancer, a potential marker for hypoxia and poor prognosis J Pathol 2012 228 434 436 10.1002/path.4100 22951989

[bib68] Zhang Y Cheng Y Ren X Zhang L Yap KL Wu H NAC1 modulates sensitivity of ovarian cancer cells to cisplatin by altering the HMGB1-mediated autophagic response Oncogene 2012 31 1055 1064 21743489 10.1038/onc.2011.290 PMC3275651

[bib69] Zhang N Qi Y Wadham C Wang L Warren A Di W FTY720 induces necrotic cell death and autophagy in ovarian cancer cells: a protective role of autophagy Autophagy 2010 6 1157 1167 20935520 10.4161/auto.6.8.13614

[bib70] Zhao S Ma CM Liu CX Wei W Sun Y Yan H Autophagy inhibition enhances isobavachalcone-induced cell death in multiple myeloma cells Int J Mol Med 2012 30 939 944 22824846 10.3892/ijmm.2012.1066

[bib71] Shingu T Fujiwara K Bögler O Akiyama Y Moritake K Shinojima N Inhibition of autophagy at a late stage enhances imatinib-induced cytotoxicity in human malignant glioma cells Int J Cancer 2009 124 1060 1071 19048625 10.1002/ijc.24030

[bib72] Liu F Liu D Yang Y Zhao S Effect of autophagy inhibition on chemotherapy-induced apoptosis in A549 lung cancer cells Oncol Lett 2013 5 1261 1265 23599776 10.3892/ol.2013.1154 PMC3628963

[bib73] Cao X Liu B Cao W Zhang W Zhang F Zhao H Autophagy inhibition enhances apigenin-induced apoptosis in human breast cancer cells Chin J Cancer Res 2013 25 212 222 23592903 10.3978/j.issn.1000-9604.2013.04.01 PMC3626985

[bib74] Gao P Bauvy C Souquère S Tonelli G Liu L Zhu Y The Bcl-2 homology domain 3 mimetic gossypol induces both Beclin 1-dependent and Beclin 1-independent cytoprotective autophagy in cancer cells J Biol Chem 2010 285 25570 25581 20529838 10.1074/jbc.M110.118125 PMC2919121

[bib75] Harhaji-Trajkovic L Vilimanovich U Kravic-Stevovic T Bumbasirevic V Trajkovic V AMPK-mediated autophagy inhibits apoptosis in cisplatin-treated tumour cells J Cell Mol Med 2009 13 3644 3654 20196784 10.1111/j.1582-4934.2009.00663.x PMC4516513

[bib76] Ren Y Huang F Liu Y Yang Y Jiang Q Xu C Autophagy inhibition through PI3K/Akt increases apoptosis by sodium selenite in NB4 cells BMB Rep 2009 42 599 604 19788862 10.5483/bmbrep.2009.42.9.599

[bib77] Filomeni G Desideri E Cardaci S Graziani I Piccirillo S Rotilio G Carcinoma cells activate AMP-activated protein kinase-dependent autophagy as survival response to kaempferol-mediated energetic impairment Autophagy 2010 6 202 216 20083895 10.4161/auto.6.2.10971

[bib78] Deng L Lei Y Liu R Li J Yuan K Li Y Pyrvinium targets autophagy addiction to promote cancer cell death Cell Death Dis 2013 4 e614 23640456 10.1038/cddis.2013.142 PMC3674351

[bib79] Henson ES Gibson SB Surviving cell death through epidermal growth factor (EGF) signal transduction pathway: implications for cancer therapy Cell Signal 2006 18 2089 2097 16815674 10.1016/j.cellsig.2006.05.015

[bib80] Kohli L Kaza N Lavalley NJ Turner KL Byer S Carroll SL The pan erbB inhibitor PD168393 enhances lysosomal dysfunction-induced apoptotic death in malignant peripheral nerve sheath tumor cells Neuro Oncol 2012 14 266 277 22259051 10.1093/neuonc/nor226 PMC3280808

[bib81] Ghadimi MP Lopez G Torres KE Belousov R Young ED Liu J Targeting the PI3K/mTOR axis, alone and in combination with autophagy blockade, for the treatment of malignant peripheral nerve sheath tumors Mol Cancer Ther 2012 11 1758 1769 22848094 10.1158/1535-7163.MCT-12-0015 PMC3416967

[bib82] Chiarini F Grimaldi C Ricci F Tazzari PL Evangelisti C Ognibene A Activity of the novel dual phosphatidylinositol 3-kinase/mammalian target of rapamycin inhibitor NVP-BEZ235 against T-cell acute lymphoblastic leukemia Cancer Res 2010 70 8097 8107 20876803 10.1158/0008-5472.CAN-10-1814

[bib83] Lin JF Tsai TF Liao PC Lin YH Lin YC Chen HE Benzyl isothiocyanate induces protective autophagy in human prostate cancer cells via inhibition of mTOR signaling Carcinogenesis 2013 34 406 414 23172666 10.1093/carcin/bgs359

[bib84] Amaravadi RK Yu D Lum JJ Bui T Christophorou MA Evan GI Autophagy inhibition enhances therapy-induced apoptosis in a Myc-induced model of lymphoma J Clin Invest 2007 117 326 336 17235397 10.1172/JCI28833 PMC1765515

[bib85] Stanton MJ Dutta S Zhang H Polavaram NS Leontovich AA Hönscheid P Autophagy control by the VEGF-C/NRP-2 axis in cancer and its implication for treatment resistance Cancer Res 2013 73 160 171 23149913 10.1158/0008-5472.CAN-11-3635 PMC3805049

[bib86] Weidhaas JB Babar I Nallur SM Trang P Roush S Boehm M MicroRNAs as potential agents to alter resistance to cytotoxic anticancer therapy Cancer Res 2007 67 11111 11116 18056433 10.1158/0008-5472.CAN-07-2858 PMC6070379

[bib87] Chen G Zhu W Shi D Lv L Zhang C Liu P MicroRNA-181a sensitizes human malignant glioma U87MG cells to radiation by targeting Bcl-2 Oncol Rep 2010 23 997 1003 20204284 10.3892/or_00000725

[bib88] Yu Y Cao L Yang L Kang R Lotze M Tang D microRNA 30A promotes autophagy in response to cancer therapy Autophagy 2012 8 853 855 22617440 10.4161/auto.20053 PMC3378424

[bib89] Yu Y Yang L Zhao M Zhu S Kang R Vernon P Targeting microRNA-30a-mediated autophagy enhances imatinib activity against human chronic myeloid leukemia cells Leukemia 2012 26 1752 1760 22395361 10.1038/leu.2012.65

[bib90] Zou Z Wu L Ding H Wang Y Zhang Y Chen X MicroRNA-30a sensitizes tumor cells to cis-platinum via suppressing beclin 1-mediated autophagy J Biol Chem 2012 287 4148 4156 22157765 10.1074/jbc.M111.307405 PMC3281695

[bib91] Xu N Zhang J Shen C Luo Y Xia L Xue F Cisplatin-induced downregulation of miR-199a-5p increases drug resistance by activating autophagy in HCC cell Biochem Biophys Res Commun 2012 423 826 831 22713463 10.1016/j.bbrc.2012.06.048

[bib92] Huang Y Chuang AY Ratovitski EA Phospho-ΔNp63α/miR-885-3p axis in tumor cell life and cell death upon cisplatin exposure Cell Cycle 2011 10 3938 3947 22071691 10.4161/cc.10.22.18107 PMC3266119

[bib93] Enzenmüller S Gonzalez P Debatin KM Fulda S Chloroquine overcomes resistance of lung carcinoma cells to the dual PI3K/mTOR inhibitor PI103 by lysosome-mediated apoptosis Anticancer Drugs 2013 24 14 19 23111416 10.1097/CAD.0b013e32835a36db

[bib94] Seitz C Hugle M Cristofanon S Tchoghandjian A Fulda S The dual PI3K/mTOR inhibitor NVP-BEZ235 and chloroquine synergize to trigger apoptosis via mitochondrial-lysosomal cross-talk Int J Cancer 2013 132 2682 2693 23151917 10.1002/ijc.27935

[bib95] Boya P Gonzalez-Polo RA Poncet D Andreau K Vieira HL Roumier T Mitochondrial membrane permeabilization is a critical step of lysosome-initiated apoptosis induced by hydroxychloroquine Oncogene 2003 22 3927 3936 12813466 10.1038/sj.onc.1206622

[bib96] Xiong HY Guo XL Bu XX Zhang SS Ma NN Song JR Autophagic cell death induced by 5-FU in Bax or PUMA deficient human colon cancer cell Cancer Lett 2010 288 68 74 19660860 10.1016/j.canlet.2009.06.039

[bib97] Lee YJ Won AJ Lee J Jung JH Yoon S Lee BM Molecular mechanism of SAHA on regulation of autophagic cell death in tamoxifen-resistant MCF-7 breast cancer cells Int J Med Sci 2012 9 881 893 23155362 10.7150/ijms.5011 PMC3498753

[bib98] Li JR Cheng CL Yang CR Ou YC Wu MJ Ko JL Dual inhibitor of phosphoinositide 3-kinase/mammalian target of rapamycin NVP-BEZ235 effectively inhibits cisplatin-resistant urothelial cancer cell growth through autophagic flux Toxicol Lett 2013 220 267 276 23651616 10.1016/j.toxlet.2013.04.021

[bib99] Cloonan SM Williams DC The antidepressants maprotiline and fluoxetine induce Type II autophagic cell death in drug-resistant Burkitt's lymphoma Int J Cancer 2011 128 1712 1723 20503272 10.1002/ijc.25477

[bib100] Leng S Hao Y Du D Xie S Hong L Gu H Ursolic acid promotes cancer cell death by inducing Atg5-dependent autophagy Int J Cancer 2013e-pub ahead of print 16 July 2013;doi:10.1002/ijc.28301 23737395

[bib101] Ren SX Shen J Cheng AS Lu L Chan RL Li ZJ FK-16 derived from the anticancer peptide LL-37 induces caspase-independent apoptosis and autophagic cell death in colon cancer cells PLoS One 2013 8 e63641 23700428 10.1371/journal.pone.0063641 PMC3659029

[bib102] Josset E Burckel H Noël G Bischoff P The mTOR inhibitor RAD001 potentiates autophagic cell death induced by temozolomide in a glioblastoma cell line Anticancer Res 2013 33 1845 1851 23645729

[bib103] Guo WJ Zhang YM Zhang L Huang B Tao FF Chen W Novel monofunctional platinum (II) complex Mono-Pt induces apoptosis-independent autophagic cell death in human ovarian carcinoma cells, distinct from cisplatin Autophagy 2013 9 996 1008 23580233 10.4161/auto.24407 PMC3722334

[bib104] Tai WT Shiau CW Chen HL Liu CY Lin CS Cheng AL Mcl-1-dependent activation of Beclin 1 mediates autophagic cell death induced by sorafenib and SC-59 in hepatocellular carcinoma cells Cell Death Dis 2013 4 e485 23392173 10.1038/cddis.2013.18 PMC3734819

[bib105] Salazar M Carracedo A Salanueva IJ Hernández-Tiedra S Lorente M Egia A Cannabinoid action induces autophagy-mediated cell death through stimulation of ER stress in human glioma cells J Clin Invest 2009 119 1359 1372 19425170 10.1172/JCI37948 PMC2673842

[bib106] Donadelli M Dando I Zaniboni T Costanzo C Dalla Pozza E Scupoli MT Gemcitabine/cannabinoid combination triggers autophagy in pancreatic cancer cells through a ROS-mediated mechanism Cell Death Dis 2011 2 e152 21525939 10.1038/cddis.2011.36 PMC3122066

[bib107] Dando I Donadelli M Costanzo C Dalla Pozza E D'Alessandro A Zolla L Cannabinoids inhibit energetic metabolism and induce AMPK-dependent autophagy in pancreatic cancer cells Cell Death Dis 2013 4 e664 23764845 10.1038/cddis.2013.151 PMC3698539

[bib108] Vara D Salazar M Olea-Herrero N Guzmán M Velasco G Díaz-Laviada I Anti-tumoral action of cannabinoids on hepatocellular carcinoma: role of AMPK-dependent activation of autophagy Cell Death Differ 2011 18 1099 1111 21475304 10.1038/cdd.2011.32 PMC3131949

[bib109] Torres S Lorente M Rodríguez-Fornés F Hernández-Tiedra S Salazar M García-Taboada E A combined preclinical therapy of cannabinoids and temozolomide against glioma Mol Cancer Ther 2011 10 90 103 21220494 10.1158/1535-7163.MCT-10-0688

[bib110] Yun SM Jung JH Jeong SJ Sohn EJ Kim B Kim SH Tanshinone IIA induces autophagic cell death via activation of AMPK and ERK and inhibition of mTOR and p70 S6K in KBM-5 leukemia cells Phytother Res 2013e-pub ahead of print 27 June 2013;doi:10.1002/ptr.5015 23813779

[bib111] Kohli L Kaza N Coric T Byer SJ Brossier NM Klocke BJ 4-Hydroxytamoxifen induces autophagic death through K-Ras degradation Cancer Res 2013 73 4395 4405 23722551 10.1158/0008-5472.CAN-12-3765 PMC3715566

[bib112] Cai Y Wan Z Sun T Shi Y Sun Y Huang P Diarylquinoline compounds induce autophagy-associated cell death by inhibiting the Akt pathway and increasing reactive oxygen species in human nasopharyngeal carcinoma cells Oncol Rep 2013 29 983 992 23291974 10.3892/or.2012.2207

[bib113] Chang HW Lee YS Nam HY Han MW Kim HJ Moon SY Knockdown of β-catenin controls both apoptotic and autophagic cell death through LKB1/AMPK signaling in head and neck squamous cell carcinoma cell lines Cell Signal 2013 25 839 847 23280187 10.1016/j.cellsig.2012.12.020

[bib114] Franzetti E Huang ZJ Shi YX Xie K Deng XJ Li JP Autophagy precedes apoptosis during the remodeling of silkworm larval midgut Apoptosis 2012 17 305 324 22127643 10.1007/s10495-011-0675-0

[bib115] Zhang N Chen Y Jiang R Li E Chen X Xi Z PARP and RIP 1 are required for autophagy induced by 11'-deoxyverticillin A, which precedes caspase-dependent apoptosis Autophagy 2011 7 598 612 21460625 10.4161/auto.7.6.15103

[bib116] Abe A Yamada H Moriya S Miyazawa K The β-carboline alkaloid harmol induces cell death via autophagy but not apoptosis in human non-small cell lung cancer A549 cells Biol Pharm Bull 2011 34 1264 1272 21804216 10.1248/bpb.34.1264

[bib117] Yahiro K Satoh M Nakano M Hisatsune J Isomoto H Sap J Low-density lipoprotein receptor-related protein-1 (LRP1) mediates autophagy and apoptosis caused by Helicobacter pylori VacA J Biol Chem 2012 287 31104 31115 22822085 10.1074/jbc.M112.387498 PMC3438942

[bib118] Francisco R Pérez-Perarnau A Cortés C Gil J Tauler A Ambrosio S Histone deacetylase inhibition induces apoptosis and autophagy in human neuroblastoma cells Cancer Lett 2012 318 42 52 22186300 10.1016/j.canlet.2011.11.036

[bib119] Amaravadi RK Lippincott-Schwartz J Yin XM Weiss WA Takebe N Timmer W Principles and current strategies for targeting autophagy for cancer treatment Clin Cancer Res 2011 17 654 666 21325294 10.1158/1078-0432.CCR-10-2634 PMC3075808

[bib120] Choi KS Autophagy and cancer Exp Mol Med 2012 44 109 120 22257886 10.3858/emm.2012.44.2.033 PMC3296807

[bib121] Buchser WJ Laskow TC Pavlik PJ Lin HM Lotze MT Cell-mediated autophagy promotes cancer cell survival Cancer Res 2012 72 2970 2979 22505650 10.1158/0008-5472.CAN-11-3396 PMC3505669

